# Evaluation of hybrid inverse planning and optimization (HIPO) algorithm for optimization in real‐time, high‐dose‐rate (HDR) brachytherapy for prostate

**DOI:** 10.1120/jacmp.v14i4.4198

**Published:** 2013-07-08

**Authors:** Shyam Pokharel, Suresh Rana, Joseph Blikenstaff, Amir Sadeghi, Bradley Prestidge

**Affiliations:** ^1^ Premiere Oncology Fort Myers FL; ^2^ Procure Proton Therapy Center Oklahoma City OK; ^3^ Arizona Radiation Oncology Specialists Scottsdale AZ; ^4^ Department of Radiation Therapy Memorial Hermanan Healthcare System Houston TX USA

**Keywords:** high‐dose rate, brachytherapy, optimization, real time, stochastic, deterministic

## Abstract

The purpose of this study is to investigate the effectiveness of the HIPO planning and optimization algorithm for real‐time prostate HDR brachytherapy. This study consists of 20 patients who underwent ultrasound‐based real‐time HDR brachytherapy of the prostate using the treatment planning system called Oncentra Prostate (SWIFT version 3.0). The treatment plans for all patients were optimized using inverse dose‐volume histogram–based optimization followed by graphical optimization (GRO) in real time. The GRO is manual manipulation of isodose lines slice by slice. The quality of the plan heavily depends on planner expertise and experience. The data for all patients were retrieved later, and treatment plans were created and optimized using HIPO algorithm with the same set of dose constraints, number of catheters, and set of contours as in the real‐time optimization algorithm. The HIPO algorithm is a hybrid because it combines both stochastic and deterministic algorithms. The stochastic algorithm, called simulated annealing, searches the optimal catheter distributions for a given set of dose objectives. The deterministic algorithm, called dose‐volume histogram–based optimization (DVHO), optimizes three‐dimensional dose distribution quickly by moving straight downhill once it is in the advantageous region of the search space given by the stochastic algorithm. The PTV receiving 100% of the prescription dose ($V_{100}$) was 97.56% and 95.38% with GRO and HIPO, respectively. The mean dose ($D_{\text{mean}}$) and minimum dose to 10% volume ($D_{10}$) for the urethra, rectum, and bladder were all statistically lower with HIPO compared to GRO using the student pair *t*‐test at 5% significance level. HIPO can provide treatment plans with comparable target coverage to that of GRO with a reduction in dose to the critical structures.

PACS number: 87.55.‐X

## I. INTRODUCTION

Radiation therapy has been a primary treatment modality for the management of adenocarcinoma of the prostate either as a sole treatment or in combination with other treatment modalities like surgery and hormonal therapy.^1^, ^2^, ^3^ The last decade has witnessed a dramatic improvement in the way radiation therapies are delivered.^(4)^ Several techniques have been developed to optimize overall treatment planning and delivery in order to increase therapeutic gain. The primary objective of treatment plan optimization is to maximize the tumor control probability (TCP) and minimize the normal tissue complication probability (NTCP) within a clinically reasonable amount of time. However, these two objectives are in conflict with each other and cannot be ideally optimized simultaneously.^(5)^ Therefore, from the practical point of view, the goal of treatment plan optimization is to provide the best compromised solution within a clinically reasonable amount of time. The treatment plan optimization as mentioned before is called a biological optimization. At present, direct biological optimization is not well‐established and is being surrogated by a physical dosimetric optimization. In dosimetric optimization, the main concern is to deliver the prescription dose (PD) to the planning target volume (PTV) accurately, keeping dose to the surrounding critical structures as low as possible to prevent treatment‐related complications. In such optimizations, biological aspects are understood to be implicitly embedded in the physician's prescription.

Different optimization algorithms have been investigated and employed successfully in HDR brachytherapy of the prostate.^6^, ^7^, ^8^ A fast simulated annealing stochastic algorithm developed by Lessard and Pouliot^(4)^ has been investigated by several groups for clinical use of HDR brachytherapy of the prostate. The overall conclusion of these investigations was that the anatomy‐based inverse treatment plan optimizations were superior to the more conventional geometrical optimization, as well as other manual optimizations.^9^, ^10^ Several other investigators have published multi‐objective optimization using dose‐volume histogram (DVH) and dose variance‐based objective functions, using both deterministic and stochastic algorithms.^7^, ^8^

Deterministic algorithms are fast, but the final result depends on the initial starting point and can be trapped in the local minima if such minima are in the objective function. It has been reported that multiple local minima may occur in radiotherapy optimization problems with dose volume constraints.^(11)^ Stochastic algorithms, such as simulated annealing (SA) or genetic algorithm (GA) are slow, but can escape from local minima and will converge to a global minimum if allowed to execute for a sufficient amount of time. In this study, we have investigated a hybrid inverse planning and optimization (HIPO) algorithm, for its efficacy in real‐time HDR brachytherapy treatment planning and optimization of the prostate. For real‐time procedures, the optimization algorithm should be fast enough to produce a clinically optimal plan within a reasonable amount of time.

## II. MATERIALS AND METHODS

This is a retrospective study consisting of data from 20 patients who underwent HDR brachytherapy to the prostate from March of 2007 to October 2009 at the Texas Cancer Clinic in San Antonio, Texas, using a treatment planning system (TPS) called Oncentra Prostate (SWIFT version 3.0). The TPS is dedicated to ultrasound based real‐time HDR brachytherapy of the prostate. The TPS is equipped with several classes of optimization algorithms, ranging from manual adjustment of dwell times to multi‐objective evolutionary inverse optimization. In addition, the TPS is equipped with pre‐implant optimization algorithms, which optimize the catheter distribution (i.e., dwell positions and dwell times) in order to create a 3D dose distribution for a given number of catheters and dosimetric constraints. The other classes of algorithms are post‐implant optimization algorithms, which optimize only the dwell times to create a 3D dose distribution for the given catheter distribution (or dwell positions), and dosimetric constraints.

The clinical plans for all patients in this study were originally optimized after catheters were implanted with the guidance of transrectal ultrasound (TRUS) based on experience. The average number of catheters used was 14 (range, 11–17), with an average prostate volume 43.27 cc (range, (23.5–67.56). Then, post‐implant optimization was carried out utilizing an inverse dose‐volume histogram–based optimization (DVHO) followed by graphical optimization (GRO) available in the TPS. Some other dose steering tools available in the TPS, such as manual adjustment of dwell times, were also used occasionally. However, the final solution was always based on GRO. In GRO, isodose lines are manually manipulated slice by slice and the TPS adjusts the dwell times accordingly. Any change in dwell times in a particular slice to create the desired dose distribution may adversely affect the neighboring slices. To reduce this effect, absolute dose mode with the local dwell times adjustment option would be useful. Nevertheless, it is a trial‐and‐error method which requires a lot of input from the user, rendering final results highly dependent on the expertise and experience of the treatment planner. In some cases it can be tedious and highly time‐consuming, which is not particularly desirable for real‐time procedures.

The primary objective of this study is to investigate an alternative treatment planning and optimization algorithm for real‐time intraoperative HDR brachytherapy of the prostate which is largely user‐independent and produces clinically optimal plans within a reasonable amount of time (3–5 minutes). The optimization algorithm chosen is the hybrid inverse planning and optimization (HIPO) which combines both stochastic (simulated annealing) and deterministic (DVH‐based) optimization algorithms. Simulated annealing (SA) is used to find the optimal catheter distribution for a given number of catheters and dosimetric constraints. Taking the SA result as an initial input, the DVH‐based inverse optimization algorithm optimizes the 3D dose distribution quickly for given dosimetric constraints. We created two plans: one with the same number of catheters as in a real‐time GRO plan which we call HIPO1, and another with three catheters less than the GRO plan which we call HIPO2. We analyzed the difference in quality of treatment plans in HIPO with the reduction in catheters. This algorithm exploits the complementary aspects of both optimization algorithms. The simulated annealing (stochastic) algorithm, which is relatively slower, is used to find the advantageous region in the search space, and the DVH (deterministic)‐based algorithm rapidly moves straight downhill to search for nearby minimum. The objective function for HIPO is given as follows:

(1) $$f_{L,PTV}(x)=\frac{1}{N_{PTV}}\sum_{i=1}^{N_{PTV}}\Theta (D_{L,PTV}-d_{i}^{PTV})(D_{L,PTV}-d_{i}^{PTV})$$

(2) $$f_{H,PTV}(x)=\frac{1}{N_{PTV}}\sum_{i=1}^{N_{PTV}}\Theta (d_{i}^{PTV}-D_{H,PTV})(d_{i}^{PTV}-D_{H,PTV})$$

Here, $f_{L,\text{PTV}}$ and $f_{H,\text{PTV}}$ are, respectively, the low‐ and high‐dose objective functions with respective dose limits of $D_{L,\text{PTV}}$ and $D_{H,\text{PTV}}$; $d_{i}^{\text{PTV}}$ is the dose value at the ith sampling point; $N_{\text{PTV}}$ is the total number of sampling points in the PTV; and *x* is the free variable for the optimization algorithm (i.e., dwell position (r) and dwell time (t) in our case). Low‐ and high‐dose objectives for the PTV ensure the conformity and homogeneity of the treatment plan. Finally, Θ(z) is the heavy side delta function defined by: 1 when z>0,½ when z=0, and 0 when z<0.

The low‐dose objective does not make sense in the case of an organ at risk (OAR), as the objective is always to achieve as low a dose as possible. In the case of OARs, only a high‐dose objective ($D_{H}$) is considered and used as follows:

(3) $$f_{H,OAR}(x)=\frac{1}{N_{OAR}}\sum_{i=1}^{N_{OAR}}\Theta (d_{i}^{OAR}-D_{H,OAR})(d_{i}^{OAR}-D_{H,OAR})$$

OARs considered in this study are the urethra, rectum, bladder, and normal tissue. The aggregate objective function is here defined by combining the individual objective functions multiplied by their respective importance or penalty factors, as follows:

(4) $$\begin{array}{l} f(x)=\sum_{i=1}^{N}w_{i}f_{i}(x)\\ f(x)=w_{l,PTV}f_{L,PTV}(x)+w_{H,PTV}f_{H,PTV}x)+\sum_{i=1}^{N_{OAR}}w_{H,OAR}f_{H,OAR}(x.) \end{array}$$

The relative importance (w) is given as $w_{i}=w_{i}/\Sigma w_{i}$, with $\Sigma w_{i}=1$. The importance factors chosen for the current study are $w_{L,\text{PTV}}=60,w_{H,\text{PTV}}=30,w_{\text{URETHRA}}=10$, and $w_{\text{BLADDER}}=w_{\text{RECTUM}}=w_{\text{NORMAL TISSUE}}=1$. These importance factors were acquired by trial and error to create an optimal plan for the first patient and then kept constant for the rest of the plans. Dose limits used for all optimizations considered were $D_{L,\text{PTV}}=100\%$ prescription dose (PD), $D_{H,\text{PTV}}=150\%(\text{PD}),D_{H,\text{URETHRA}}=120\%(\text{PD}),D_{H,\text{RECTUM}}=85\%(\text{PD}),D_{H,\text{BLADDER}}=85\%(\text{PD})$, and $D_{H,\text{NORMAL TISSUE}}=150\%(\text{PD})$.

### A. Execution of HIPO

**Figure 1:**
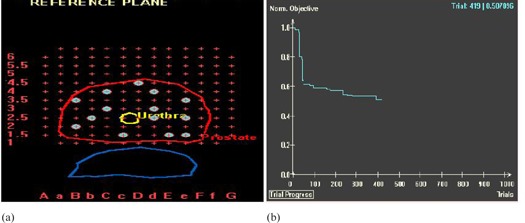
HIPO starts with a simulated annealing algorithm to find the optimal catheter distribution for a given anatomy and user defined number of catheters, objectives, dosimetric constraints and penalties. As trials progress, HIPO (a) places a catheter in a feasible but unoccupied template hole randomly and the superiority of the resulting catheter distribution is tested based on the aggregate objective function (b) normalized to its initial (first trial) value stochastically. If the user does not interrupt, the algorithm runs for the predefined number of trials.

The HIPO algorithm starts with a user defined number of catheters placed at random in all feasible template holes within the projection of the template on the reference slice, as shown in Fig. 1(a). The user can define the margin to any contoured structures in order to let the TPS know the feasible template holes. In this study, 0 mm and 5 mm margins were set for the prostate and urethra, respectively. So, template holes 5 mm away from the urethra and on or within the prostate contour are deemed feasible.

The algorithm changes one of the catheters at random to another unoccupied feasible position, and the resulting catheter distribution is accepted or rejected based on the aggregate objective function normalized to its initial (first trial) value, stochastically using the simulated annealing algorithm, as shown in Fig. 1(b). If the user does not interrupt, the algorithm runs for a predefined number of trials. If a plateau is achieved in a normalized total objective function curve for at least 200 trials, it is an indication that further improvement in dose distribution for given dose limits and penalties is not expected with the change in catheter geometry. In this study, we never had to run more than a total of 600 trials to arrive at optimal catheter distribution.

**Figure 2:**
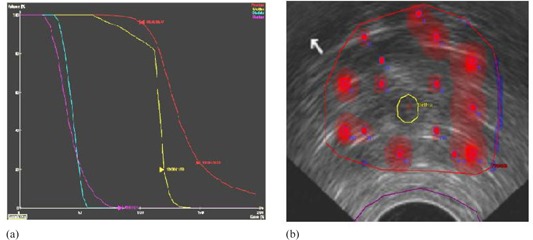
Once HIPO finds an optimal catheter distribution (a) using the simulated annealing algorithm, it then takes this as an initial input. Then the DVHO algorithm optimizes the 3D dose distribution for the given catheter distribution, objectives, dosimetric constraints, and penalties deterministically. DVHO algorithm (b) is guided to generate an ideal DVH and tries to reduce hot areas (red), or cold areas (blue) in the case of the PTV, and tries to reduce the upper dose limit for each OARs deterministically.

Once the simulated annealing part of HIPO finds the optimal catheter distribution, then the DVH‐based optimization algorithm optimizes the 3D dose distribution for the given aggregative objective function with given penalties and dose limits within a few seconds. The DVHO algorithm tries to reduce the volume receiving upper and lower dose limits in the case of the PTV, and reduces the volume receiving upper dose limit in the case of OARs iteratively, as shown in Fig. 2. As the DVHO algorithm starts from this advantageous region in search space, determined by the simulated annealing algorithm, it is expected (though not guaranteed) that the DVHO algorithm converges to a global or near global minimum.

### B. Dosimetric evaluation and comparison

**Figure 3:**
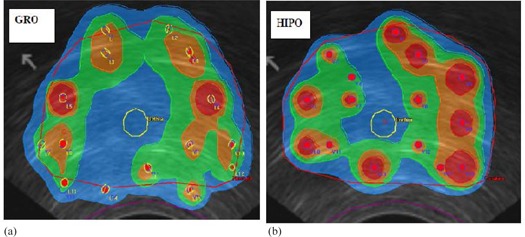
A comparison of the isodose distribution in colorwash form between GRO (a) and HIPO (b) for the same axial slice. The PTV is enclosed with a red contour, blue=100% isodose, green=125%,brown=150%,light coral=200%. Normal tissue treated outside the PTV is less with HIPO than with GRO.

**Figure 4:**
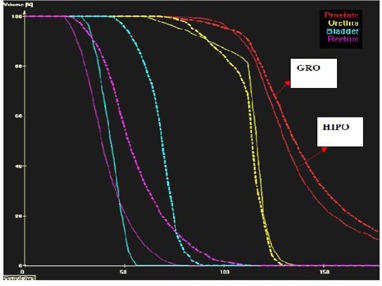
A DVH comparison between GRO (dotted lines) and HIPO (solid lines) for a typical case. HIPO yields smaller dose to the bladder and rectum compared to GRO with comparable dose to urethra and prostate.

Isodose distribution and several dosimetric quality indices obtained from cumulative DVH were used for qualitative, as well as quantitative, comparison of different treatment plans optimized by different optimization algorithms. Figure 3 shows the isodose distribution comparison of the same axial slice of a particular patient between GRO and HIPO1. Figure 4 shows the cumulative DVH comparison between treatment plans optimized by GRO and HIPO1 of the same patient. The following are the dosimetric quality indices calculated to compare treatment plans quantitatively:

1. $D_{90}$ — the dose that covers 90% of PTV.
2. $V_{100},V_{150}$, and $V_{200}$ — the volume of PTV receiving 100%, 150%, and 200% of the PD, respectively.
3. $D_{10}$ and $D_{\text{mean}}$ of OARs — $D_{10}$ is the minimum dose to 10% of the OAR volume (urethra, bladder or rectum); $D_{\text{mean}}$ is the mean dose to a given volume of an OAR.
4. Homogeneity Index (HI) — defined as $\text{HI}=(V_{100}-V_{150})/V_{100}$; this index is used to assess the volume of hot spot generated relative to the treatment volume.
5. Conformal Index (COIN) — a unique quality index that describes how well the reference isodose covers the target volume and excludes nontarget volumes.^(12)^ It is defined as:

(5) $$\text{COIN}=({\text{PTV}}_{\text{ref}}/\text{PTV})\times ({\text{PTV}}_{\text{ref}}/V_{\text{ref}})$$

where ${\text{PTV}}_{\text{ref}}$ is the volume of PTV that receives dose equal to or greater than PD; $V_{\text{ref}}$ is the volume receiving the PD. The ideal situation is that in which COIN is equal to 1. In real clinical situations it is always less than 1 and, if all other parameters are comparable, then a treatment plan with higher COIN should be favored.

### C. Statistical analysis

The student pair *t*‐test at 5% level of significance was used to make statistical comparisons of different dosimetric quality indices of treatment plans optimized by different optimization algorithms. The statistical comparisons were carried out between HIPO1 vs. GRO, HIPO1 vs. HIPO2, and HIPO1 vs. DVHO. The comparison between HIPO and DVHO with same weighting factors was just carried out to signify the importance of dwell position optimization.

## III. RESULTS

**Figure 5:**
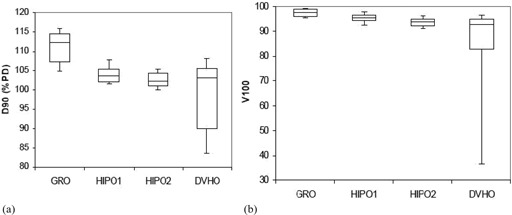
Box and whisker plots of the $\text{PTV}\;D_{90}(a)$ and $V_{100}$ (b) for the four optimization algorithms covered in this study. DVHO has the widest range of $D_{90}$ and $V_{100}$ rendering clinically unacceptable most of the time. The plots present the 10th percentile, 25th percentile, median, 75th percentile, and 90th percentile of data used.

**Figure 6:**
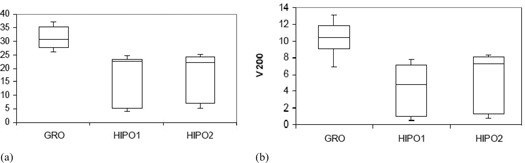
Box and whisker plots of the $\text{PTV}\;V_{150}(a)$ and $V_{200}$ (b) for GRO, HIPO1, and HIPO2. Both are significantly larger with GRO.

**Figure 7:**
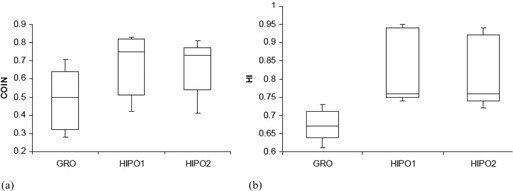
Box and whisker plots of COIN (a) and HI (b) for GRO, HIPO1, and HIPO2. Both are bigger with HIPO1 as compared to GRO. HIPO2 has comparable COIN and HI to that of HIPO1.

**Figure 8:**
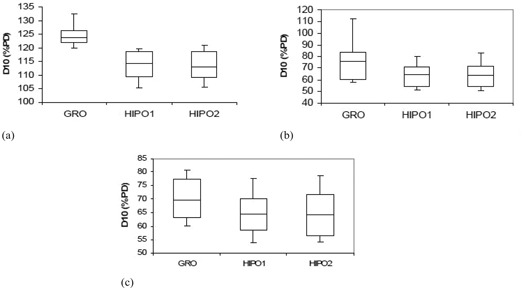
Box and whisker plots of $D_{10}$ for critical structures urethra (a), bladder (b), and rectum (c) for GRO, HIPO1, and HIPO2. All are greater with GRO compared to HIPO1. There is no significant change in $D_{10}$ for each structure between HIPO2 and HIPO1.

**Figure 9:**
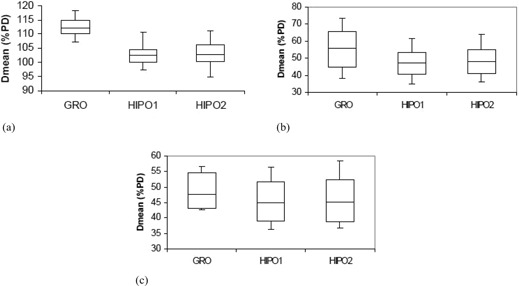
Box and whisker plots of $D_{\text{mean}}$ for the urethra (a), bladder (b), and rectum (c) for GRO, HIPO1, and HIPO2. Mean doses to all critical structures are larger with GRO compared to HIPO1 and are comparable between HIPO1 and HIPO2.

The statistical analysis of dosimetric quality indices for both PTV and OARs is presented in Tables 1 and 2, respectively. The mean $D_{90}$ and $V_{100}$ for the GRO optimized plans used to treat the patients in real‐time are 110.88% (range, 102.43%–119.50% of the PD) and 97.56% (range, 94.04%–99.72%), respectively. The PTV $D_{90}$ with GRO is significantly larger (p<0.001) compared with HIPO1 for comparable $V_{100}$, as presented in Table 1 and Fig. 5. The $V_{150}$ and $V_{200}$ are both significantly bigger in plans optimized by GRO, as compared to plans optimized by HIPO1 (Table 1 and Fig. 6). Both HI and COIN are significantly higher in plans optimized by HIPO1 compared to plans optimized by GRO (Table 1 and Fig. 7). The slight decreases in $D_{90},V_{100}$, COIN, and HI and slight increases in $V_{150}$ and $V_{200}$ are observed in the plans optimized with HIPO2 compared to plans optimized with HIPO1 (Table 1 and Figs. 5 to 7). The $D_{10}$ for the critical structures urethra, bladder, and rectum are all statistically greater in the plans optimized by GRO compared to plans optimized by HIPO1 (Table 2 and Fig. 8). Similarly, $D_{\text{mean}}$ for all the critical structures are statistically larger in the plans optimized by GRO compared to plans optimized by HIPO1 (Table 2 and Fig. 9).

**Table 1:** Dosimetric indices for the PTV with different optimization algorithms

*Optimization Algorithm*	$D_{90}(\%\;\text{PD})$	$V_{100}(\%\;\text{PD})$	$V_{150}(\%\;\text{PD})$	$V_{200}(\%\;\text{PD})$	*COIN*	*HI*
GRO						
Mean	110.88	97.56	31.94	10.46	0.50	0.67
σ	4.85	1.77	7.31	2.69	0.18	0.07
Minimum	102.43	94.04	19.87	6.26	0.19	0.48
Maximum	119.50	99.72	50.10	17.50	0.81	0.79
HIPO1						
Mean	103.98	95.38	16.45	4.88	0.67	0.83
σ	2.46	1.88	9.00	3.17	0.18	0.09
Minimum	100.24	92.11	3.82	0.19	0.25	0.73
Maximum	109.57	98.15	25.40	8.06	0.86	0.96
p‐value	<0.001	0.001	<0.001	<0.001	0.015	<0.001
HIPO2						
Mean	102.55	93.93	17.58	5.56	0.65	0.81
σ	2.26	2.00	8.70	3.42	0.17	0.09
Minimum	98.87	90.73	4.26	0.50	0.25	0.69
Maximum	107.30	96.76	28.73	9.89	0.86	0.95
p‐value	<0.001	<0.001	0.008	<0.001	0.102	0.001
DVHO						
Mean	97.46	82.66	15.05	4.40	0.49	0.82
σ	11.31	21.60	8.93	3.39	0.19	0.10
Minimum	71.13	32.32	2.11	0.60	0.12	0.58
Maximum	108.81	97.38	35.11	11.70	0.81	0.93
p‐value	0.01	0.02	0.670	0.705	0.015	0.910

PTV= planning target volume; PD=prescription dose; GRO=graphical optimization; HIPO1= hybrid inverse planning and optimization with same number of catheters as in GRO; HIPO2= three catheters less than HIPO1; σ=standard deviation; DVHO= dose‐volume histogram–based optimization; HI=Homogeneity Index; D90= minimum dose to 90% of the PTV; $V_{100},V_{150}$, and $V_{200}=$ volume receiving 100%, 150% and 200% of PD, respectively.

**Table 2:** Dosimetric indices for the OARs with different optimization algorithms

*Optimization Algorithm*	$D_{10}$ *(Urethra)*	$D_{\text{mean}}$ *(Urethra)*	$D_{10}$ *(Bladder)*	$D_{\text{mean}}$ *(Bladder)*	$D_{10}$ *(Rectum)*	$D_{\text{mean}}$ *(Rectum)*
GRO						
Mean	127.09	113.47	76.47	55.80	69.40	48.99
σ	13.54	8.05	20.26	14.53	8.77	7.03
Minimum	117.01	101.98	51.18	36.17	50.32	36.52
Maximum	181.94	141.84	118.96	87.66	85.66	62.18
HIPO1						
Mean	113.46	102.39	64.62	47.78	64.55	45.35
σ	6.30	6.18	12.81	10.59	9.50	8.36
Minimum	99.56	88.22	46.05	32.22	44.85	30.21
Maximum	123.47	112.85	92.27	72.66	80.62	60.28
p‐value	<0.001	<0.001	0.006	0.008	0.022	0.022
HIPO2						
Mean	113.30	102.75	65.15	48.55	64.57	45.59
σ	6.62	6.01	12.21	10.11	10.79	8.36
Minimum	99.08	88.12	47.60	33.11	44.41	30.10
Maximum	124.33	112.87	91.61	67.03	85.53	60.28
p‐value	0.783	0.507	0.474	0.271	0.031	0.529
DVHO						
Mean	109.10	98.56	74.26	53.86	70.53	49.92
σ	13.67	13.00	24.71	17.49	12.25	9.09
Minimum	76.20	69.09	34.65	24.72	53.54	37.04
Maximum	121.85	113.28	112.70	84.24	98.08	67.16
p‐value	0.183	0.233	0.094	0.157	0.743	0.097

D10= minimum dose to 10% of urethra, bladder, or rectum volume; $D_{\text{mean}}=$ mean dose to a given volume of interest.

## IV. DISCUSSION

The treatment plans for the cohort of patients in this study were optimized with inverse DVHO algorithm followed by GRO algorithm in real time. DVHO algorithm attempts to reduce hot and cold spots within the PTV, and tries to reduce the upper dose limit to the critical structures in an effort to create an ideal DVH. Because the algorithm is deterministic, it is a very quick optimization algorithm and moves straight downhill to a nearby minimum. The catheters for real‐time procedures were implanted manually, based on the experience of the planners, so as to have adequate PTV coverage while maintaining adequate protection of critical structures. The post‐implant optimization was carried out by a DVHO algorithm for the aggregate objective function, as given in Eq. (4), with dose constraints, as previously mentioned. The weighting factors for the DVHO algorithm were optimized for the first clinical case, then defaulted for the rest of the plans optimized. The set of importance factors for clinical DVHO algorithm was different than one previously given for HIPO algorithm. Most of the time, plans were not clinically acceptable without further improvement utilizing the dose steering tools available in the TPS. Clinically acceptable plans include PTV coverage of at least 90% PD while meeting nontarget tissue constraints. It is always possible to improve the treatment plans by repeating the optimization with different sets of importance factors using trial and error until the planner considers the optimization result acceptable. We, however, did not try that approach; instead the plans were further optimized using GRO. GRO is the optimization method in which isodose lines are manipulated on the computer screen slice by slice as desired, and the TPS adjusts the dwell times accordingly. Manipulation of isodose lines in one slice may deteriorate the isodose distribution in the neighboring slices, and the planner has to continue manipulating the isodose distribution until the plan is considered clinically acceptable. The final plan given by GRO heavily depends on the expertise and experience of the planner and it sometimes requires more time, which is not desirable in real‐time procedures.

In an attempt to find an alternative planning and optimization algorithm which is robust, less user dependent, and still produces a clinically optimal plan within a clinically reasonable amount of time, a hybrid type of inverse planning and optimization algorithm is chosen for the present study. HIPO combines a stochastic algorithm (SA) and a deterministic algorithm (DVHO). The SA searches for an optimal catheter distribution for a given set of objectives and the DVHO optimizes the 3D dose distribution once in this advantageous region of the search space. We observed a huge improvement in treatment plans optimized by DVHO with optimal catheter distribution determined by simulated annealing to that of DVHO optimization with manual catheter implantation for the same dose limits, number of catheters, and importance factors. All of the HIPO created and optimized plans met minimum clinical acceptability criteria in our clinic. Once we have the anatomical information, along with intended number of catheters, it takes 3 to 5 minutes for both planning and optimization. The average time to carry out HIPO in this study was 3.8 minutes. The manual optimization for a typical experienced brachytherapy team takes about 20–35 minutes. Significant time is expected to be saved during the catheter implantation process, as well, because the physician and physicist already know where to implant the needles.

HIPO1 has slightly less $V_{100}$ (97.56% vs. 95.38%) to that of GRO, but with a significant increase in COIN and HI (Table 1). The $D_{90}$ (110.88 vs. 103.98) for HIPO1 is significantly smaller than with GRO, mostly due to significant decreases in $V_{150}$ (31.94 vs. 16.45) and $V_{200}$ (10.46 vs. 4.88). However, there is no established relationship between $D_{90}$ and clinical outcome in HDR brachytherapy, as in the case of low‐dose‐rate permanent seed implantation. So, to date, all we can tell is that as long as $D_{90}$ is more than 100% of PD, the plan can be considered clinically acceptable, provided other dosimetric quality indices, including $V_{100}$, are acceptable. The $D_{90}$ with HIPO1 in this study was always greater than 100% of the PD (range, 100.24%–109.57%).

A smaller number of catheters are desirable to reduce prostatic trauma, edema, and possible displacement. We created HIPO2 optimized plans which have three catheters less than HIPO1, keeping all other parameters fixed. HIPO2 plans have essentially the same dosimetric quality indices for all OARs (Table 2) with a slight decrease in coverage parameters ($D_{90}$ and $V_{100}$, Table 1). It appears that the HIPO algorithm best manages the given number of catheters and is reasonably immune to a small variation in catheter numbers.

## V. CONCLUSIONS

HIPO is a unique treatment planning and optimization tool, capable of producing clinically acceptable treatment plans within a clinically reasonable time (3–5 minutes) for real‐time intraoperative HDR brachytherapy of the prostate. We have found that plans created by the HIPO algorithm are comparable, if not better, than the plans created by an experienced user utilizing manual planning and optimization. The HIPO algorithm provides an optimal catheter distribution based on anatomy to best realize the user's defined objectives within given dose limits and penalties. With HIPO, the overall treatment planning and optimization time is expected to significantly reduce. GRO is an intuitive optimization tool and can always produce clinically acceptable plans, but the overall optimization time and the final quality of the plan heavily depends on planner expertise and experience. GRO is extremely useful to make final fine‐tune adjustments, but can be tedious and extremely time‐consuming, in some cases, if most of the optimization is performed using this tool.
